# The Epidemiological and Clinical Findings from the Latvian Registry of Primary Congenital Glaucoma and Evaluation of Prognostic Factors

**DOI:** 10.3390/medicina57010044

**Published:** 2021-01-07

**Authors:** Eva Elksne, Kristine Baumane, Arturs Ozolins, Sandra Valeina

**Affiliations:** 1 Department of Ophthalmology, Riga Stradins University, LV-1007 Riga, Latvia; Sandra.Valeina@bkus.lv; 2 Department of Ophthalmology, Clinical Centre Bikernieki, Riga East University Hospital, LV-1006 Riga, Latvia; Baumanek@ml.lv; 3 Faculty of Medicine, University of Latvia, LV-1586 Riga, Latvia; 4 Department of Surgery, Pauls Stradins Clinical University Hospital, LV-1002 Riga, Latvia; Arturs.Ozolins@stradini.lv; 5 Faculty of Medicine, Riga Stradins University, LV-1007 Riga, Latvia; 6 Department of Ophthalmology, Children’s Clinical University Hospital, LV-1004 Riga, Latvia; 7 European Reference Network on Rare Eye Diseases (ERN-EYE), Children’s Clinical University Hospital, LV-1004 Riga, Latvia

**Keywords:** primary congenital glaucoma, primary paediatric glaucoma, incidence, clinical findings, surgical treatment

## Abstract

*Background and objectives*: primary congenital glaucoma (PCG) is a rare, potentially blinding disease that affects children worldwide. The aim of the study was to describe the epidemiological and clinical characteristics, outcomes for newly diagnosed patients with PCG, as well as evaluate the prognostic factors that are related to the outcomes. *Materials and Methods*: a retrospective cohort study was conducted at a tertiary referral centre among patients diagnosed with PCG. Evaluation of the clinical data was performed preoperatively at three, six, and 12 months after the surgery and at the last follow-up. *Results*: during the 15 years of follow-ups, 24 eyes of 18 patients were diagnosed with PCG. Unilateral and bilateral PCG constituted 50% of cases each. A slight male predominance was observed (55.6% vs. 44.4%), with a relative risk of 1.3. The incidence of PCG was 1:19,033 live births. The mean age of the patients at the time of diagnosis was 10.1 ± 10.0 months, with a diagnostic delay of 2.0 ± 1.9 months. Furthermore, 75% of patients indicated an enlargement of an eyeball, followed by excessive tearing (58.3%) and corneal opacity (41.7%). After 85.9 ± 51.2 months, the mean intraocular pressure (IOP) value was 14.6 ± 4.9 mmHg. Surgical treatment provided sufficient IOP control in 75% of PCG cases at the last follow-up visit. The only prognostic factor that was related to the outcome of IOP control that was statistically significant was axial length at the time of diagnosis. *Conclusions*: the incidence of PCG in Latvia was 5.3 patients per 100,000 live births. PCG was more common among males than females with a relative risk of 1.3. The enlargement of an eyeball was the leading clinical sign.

## 1. Introduction

Primary congenital glaucoma (PCG) is a rare, potentially blinding disease that affects children worldwide [1,2]. The pathogenesis of the entity relies on an isolated dysgenesis of the anterior chamber angle that causes increased intraocular pressure (IOP) and, subsequently, optic nerve head (ONH) damage, leading to an irreversible loss of vision [3]. Most of the cases are diagnosed within the first year of life, and population-based statistics reflect a slight male gender predominance [4].

The goal of the treatment is to reduce IOP and, by the application of this approach, protect the retinal ganglion cells from further damage [5]. The gold standard is surgical treatment that can include goniotomy or trabeculotomy and, in resistant cases, trabeculectomy and implantation of tube shunts [6].

Population-based prospective studies are relatively complicated in the field of PCG, due to the rare nature of the disease. Thus, a retrospective approach is often applied. Until now, there are no reported data for PCG in Latvia. Several publications that are related to the incidence of PCG in European countries are available, thus increasing interest in the incidence in the Latvian population [1,7,8].

The aim of the study was to describe the epidemiological and clinical characteristics, and the results of the treatment of newly diagnosed patients with PCG from 2003 to 2017, as well as to evaluate the prognostic factors that are related to the outcomes among the population of Latvia.

## 2. Materials and Methods

A retrospective cohort study was conducted among consecutive patients that were diagnosed with PCG and treated at a tertiary medical centre. The study lasted from 1 January 2003 to 31 December 2017. The study was approved by the local ethics committee of Riga Stradins University (the ethical approval number 9/25.09.2017.) and all of the data were collected according to the guidelines on data protection and confidentiality.

The research included an analysis of the clinical data of the medical files that were related to diagnosis (Q15.0).

The inclusion criteria for the study were set according to the definition of PCG standardized by Childhood Glaucoma Research Network. Two or more criteria were required in order to diagnose PCG: IOP >21 mm Hg, optic disc cupping (progressive increase in cup-disc ratio, cup-disc asymmetry of 0.2 when optic discs are of similar size, and focal rim thinning), corneal findings (Haab striae, diameter >11 mm in newborn, >12 mm in children less than one year of age or >13 mm in older than one year), ocular enlargement by progressive myopia, or axial length out of keeping with normal growth, or a visual field defect that is consistent with glaucoma [9]. The age of onset was classified after the guidelines of European Glaucoma Society, 4th edition: neonatal or newborn onset (0–1 months), infantile onset (1–24 months), and late onset or late recognized for children after the age of 24 months [10].

The exclusion criteria were, as follows: any secondary childhood glaucoma associated with other congenital abnormalities or comorbidities.

The Department of Ophthalmology of Children’s Clinical University Hospital in Riga is the only institution in the country that deals with PCG. During the study period, all of the children from Latvia with typical symptoms and signs of PCG were referred to our clinic. Experienced specialists performed the full examination and treatment. The diagnostic procedures were performed according to the PCG protocol of the department. Examination data included the value of IOP (mmHg) at an early phase of general anaesthesia, horizontal corneal diameter (mm), axial length measurement (mm), biomicroscopic examination of anterior segment, gonioscopy, and indirect fundoscopy.

Clinical data analysis also included a study of family history, age when the onset of the symptoms appeared, age when diagnosis was stated, and diagnostic delay. The age at the appearance of symptom was defined when the signs of PCG (for example, excessive tearing, photophobia, and enlargement of an eyeball) were noticed for the first time by parents, guardians, or others. The age at diagnosis was set when a doctor diagnosed the disease for the first time.

All of the participants underwent surgical treatment. As a result of the low number of patients with PCG per year and high-level of experience in performing trabeculectomies, all of the children received mitomycin C (MMC) 0.4 mg/mL-augmented trabeculectomy as the primary treatment. None of patients received goniotomy or trabeculotomy. After the surgery, topical glaucoma medications were added stepwise in cases in which an increase in IOP was observed.

The patients were monitored at three, six, and 12 months after the surgery and about once a year thereafter in the tertiary medical centre, thus providing long-term retrospective data after the primary treatment. Every consultation included a determination of visual acuity (with the most appropriate method according to the patient’s age), objective refraction (retinoscopy), measurement of IOP, and an evaluation of the clinical signs.

The demographic data regarding live-born children in the population during the same period were obtained from an online national database (Central Statistical Bureau of Latvia).

### Statistical Data Analysis

For normally distributed data, the mean and standard deviations were utilised; otherwise, the median and interquartile ranges were used. Categorical data were reflected as counts and percentages.

Incidence was calculated by the total number of newly diagnosed cases during the retrospective study, divided by the number of live births during the same period. The same criterion was applied for gender-related incidence. A paired-sample *t*-test was applied in order to observe the effect of surgical treatment on IOP. A *p* value that was below 0.05 was considered to be statistically significant. One-way analysis of variance (ANOVA) was applied to evaluate the association between different factors and surgical outcome. 

The data were analysed using statistical software (IBM Corp. Released 2016. IBM SPSS Statistics for Macintosh, Version 24.0. IBM Corp, Armonk, NY, USA).

## 3. Results

**Figure 1:**
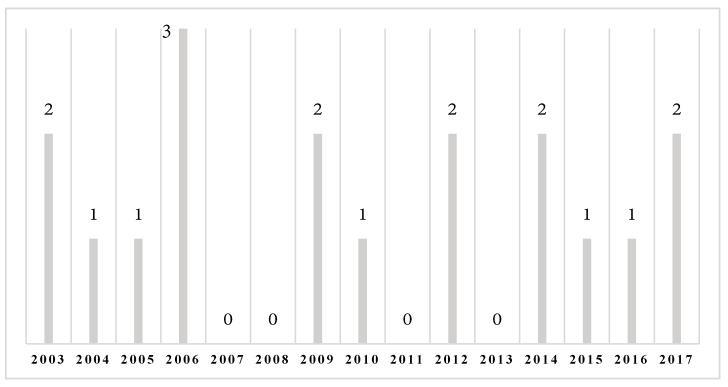
Number of patients per year diagnosed with primary congenital glaucoma (PCG).

During the 15 years of follow-ups, 24 eyes of 18 patients were diagnosed with PCG (Figure 1). Unilateral and bilateral cases constituted 50% each. A slight male predominance was observed (55.6% vs. 44.4%). All of the patients were of Caucasian decent, and no consanguinity was confirmed. Only one case (5.6%) indicated positive family anamnesis.

From 2003 to 2017, the live birth rate in Latvia was 342,598 children in total. Consequently, during the study period, the incidence of PCG was 1:19,033 live births (5.3 patients per 100,000). The incidence in the male population was 5.7 per 100,000 live births and, in the female population, it was 4.8 per 100,000 (Table 1). The relative risk for a male was 1.3.

### 3.1. Age at Diagnosis and Diagnostic Delay

The mean age at the time of diagnosis was 10.1 ± 10.0 months (ranging from 0.5 to 44 months). A comparison of unilateral and bilateral cases revealed mean age differences (6.6 ± 2.8 vs. 13.8 ± 13.0). The mean value of diagnostic delay was 2.0 ± 1.9 months (range 0 to 6 months). Table 2 portrays the distribution of patients, according to the age at defining diagnosis and diagnostic delay. Most of the patients were diagnosed between six and 12 months of age and, until the end of the first year of life, 94.4% of the cases were diagnosed as having PCG. More than a half of patients were diagnosed with PCG within three months of the appearance of first symptoms and signs.

### 3.2. Signs and Symptoms

For 75% of patients, the enlargement of an eyeball (increase of the palpebral aperture and/or anterior segment of the eye) was noticed by parents or guardians. It was followed by excessive tearing (58.3%) and corneal opacity (41.7%), as described in Table 3.

At the time of diagnosis, the median corneal diameter was 12.5 mm. The mean axial length was 22.4 ± 2.3 mm. Furthermore, IOP was 27.4 ± 4.1 mmHg.

### 3.3. The Surgical Outcome

**Figure 2:**
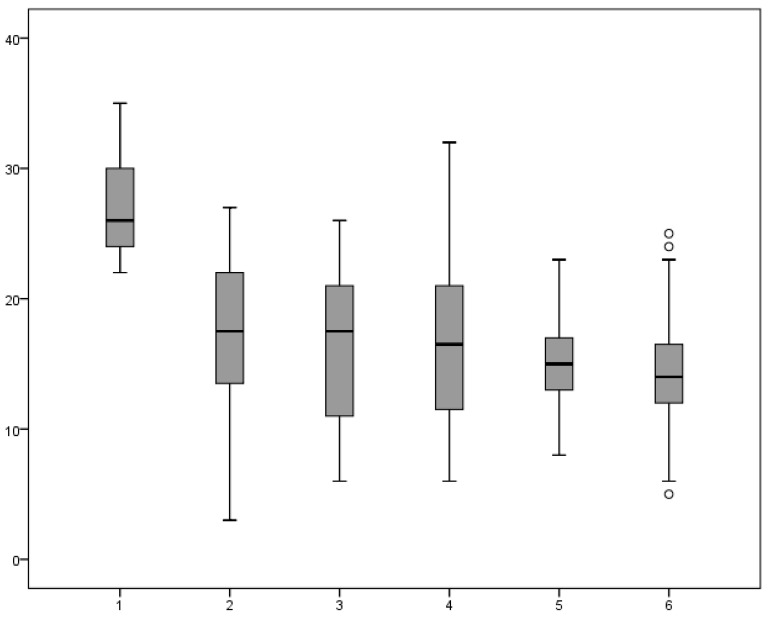
Intraocular pressure (IOP) reduction after the surgery and values during follow-up. 1—before surgery, 2—after surgery, 3—after three months, 4—after six months, 5—after 12 months, 6—at the last follow-up.

Trabeculectomy was the only surgical treatment and none of the patients received repeated surgery during the follow-up (85.9 ± 51.2 month). The surgical outcome (Figure 2) was evaluated at the day of discharge, after three, six, and 12 months, and at the last follow-up.

The last follow-up revealed a stable reduction in IOP, with its value reaching of 14.6 ± 4.9 mmHg (Table 4). If an increase in IOP was noticed, then topical glaucomatous medication was administered gradually. 

At the last follow-up, 75% of the eyes reached complete success (IOP ≤21 mmHg without glaucoma medications), and 12.5% were described as a qualified success (IOP ≤21 mmHg managed by topical antiglaucomatous drugs). However, in 12.5% of the eyes, the treatment was classified as having failed because the control of IOP had not been achieved.

### 3.4. Visual Acuity and Refractive Error

At the last follow-up, the mean visual acuity, according to LogMAR scale, was 0.5 ± 0.4. The spherical equivalent reached −1.8 ± 3.0 dioptres (D). Myopia and astigmatism were diagnosed in 37.5% (−1.8 ± 1.8 D) and 75.0% (−1.6 ± 1.4) of patients. Amblyopia was diagnosed in 15 (62.5%) of eyes.

### 3.5. Prognostic Factors Related to the Outcome

Axial length at the time of diagnosis (r^2^ = 0.40 *p* = 0.01) was the only prognostic factor related to the outcome of IOP control that was statistically significant. No statistically significant level was found between the surgical outcome at the last follow-up and the patient’s age (*p* = 0.12), gender (*p* = 0.52), laterality of the disease (*p* = 0.90), enlargement of the eyeball (*p* = 0.28), epiphora (*p* = 0.07), photophobia (*p* = 0.20), corneal diameter (*p* = 0.20), IOP before surgery (*p* = 0.48), or diagnostic delay (*p* = 0.48).

## 4. Discussion

The report describes the incidence of PCG in Latvia during a 15-year period. Until now, it is the first data collection that is related to the disease, thus making a comparison with other countries possible. Furthermore, the report describes the typical symptoms and clinical signs that are related to the disease, providing data regarding the impact of diagnostic delays and possible improvements to the diagnostic approach towards PCG. As it is a potentially blinding disease with a huge impact to quality of life—it accounts for 5% of child blindness, an early, precise diagnosis and effective treatment are the principal objectives [11].

The incidence among the population in Latvia was 5.3 patients per 100,000 live born. The data that were obtained in the scope of this study are in accordance with studies from other parts of Europe, such as Great Britain (5.41), England (5.13), Sweden (4.3), and Denmark (4.8) [1,7,12]. Table 5 reflects the incidence of PCG in various countries.

By 2017, the total population of Latvia was 1.95 million. The mean live birth rate over the 15 years proceeding this study was about 22,840 per year. As was reflected in the present study, the incidence of new cases per year ranged from 0 to 3, showing PCG to be a very rare disease and demonstrating the problems in terms of diagnosis. In comparison to other Western countries, as a result of the small population, all of the patients were treated in one clinical centre by the same doctors.

Previous publications cited that the mean incidence of PCG in Western countries ranges from 1 in 10,000–20,000 to 1 in 68,000 live births [1,15,18]. Higher rates have been registered among Slovakian gypsies one in 1250) and in Saudi Arabia (one in 2500), suggesting consanguinity as the possible mechanism [14,17].

The present study affirmed a slight dominance of the male gender in terms of higher relative risk of developing PCG (1.3 in comparison to females), which is in accordance with previous studies [4,7]. Despite bilateral PCG being the leading form of the disease, unilateral and bilateral PCG each constituted 50% of cases [14,19,20]. However, the relatively small number of patients taking part in this study should be considered to be a possible weakness. 

The mean age at the time of diagnosis was 10.1 ± 10.0 months, which is in agreement with the study conducted by Aziz et al. [8]. However, several publications have claimed that, for most patients, PCG is diagnosed before the age of six months [1,7,12,21]. In the scope of the present study, this was mainly between six and 12 months. Diagnostic delay, which was 2.0 ± 1.9 months, could have affected this outcome. Furthermore, the rare incidence of the disease and misleading symptoms could have exacerbated the diagnostic delay among the research population. 

The typical symptoms of PCG are tearing, photophobia, and blepharospasm. However, in most of the cases—including those in this study—the enlargement of an eyeball is the leading diagnostic sign of PCG [21]. Tearing was the second most common symptom, but this usually caused diagnostic problems, as possible nasolacrimal duct stenosis need to be excluded before a positive PCG diagnosis could be made. Thus, there was a prolonged diagnostic delay for these patients. PCG is quite rare among the population of Latvia; therefore, the first symptoms are not always related to a primary congenital anterior chamber anomaly. It takes time for ocular enlargement and corneal opacity to develop as a response to a prolonged increase in IOP. Conducting a proper examination of infants and new-borns without general anaesthesia is still the main problem for PCG. 

At the time of diagnosis, the median corneal diameter was 12.5 mm and axial length was 22.4 ± 2.3 mm. Both of the values are significantly increased in comparison to data on normal measurements for children before the age of 12 months. A corneal diameter larger than 11 mm for a new-born and above 12 mm for a child less than one year is pathological [1]. Central corneal thickness (CCT) was not considered in this study. Furthermore, according to Childhood Glaucoma Research Network, CCT should not be applied to adjust IOP measurements, as its utility in childhood tonometry remains to be determined [9]. The normal axial length for a new-born is about 16.8 mm and about 20.6 mm for a 12-month-old child [22,23].

After 85.9 ± 51.2 months, a stable IOP reduction was observed without a statistically significant difference during the follow-up period. Currently, the reduction in IOP is the only approach for preserving the visual function of children with PCG. At the last follow-up, 87.5% of the patients reached IOP ≤ 21 mmHg. This suggests that most of the patients presented a controlled IOP. However, an ab-externo approach is in agreement with the possible increase in astigmatism, which was diagnosed in 75% of patients. Other publications have evaluated the success rate of trabeculectomy as the primary approach for PCG, with a range from 54% to 90% [24,25]. In our study, patients only received MMC-augmented trabeculectomy as a result of the low number of patients with PCG per year and high-level of experience in performing trabeculectomies in children. According to guidelines, it is not the first line treatment for PCG and it is reserved when goniotomy or trabeculotomy fail. However, in our population, trabeculectomy until now is the only option for surgical treatment of PCG. Performing glaucoma surgeries for children is very challenging and requests to have appropriate training while the learning curve could significantly affect the outcome. The low number of cases per year affects the introduction of different surgical techniques and it is mandatory that the surgeon is confident with the manipulation. Our national experience reflects that this type of treatment is acceptable, as it provides the control of IOP in most cases during 15 years of follow-ups.

Amblyopia was diagnosed in more than a half of all the eyes, and this remains a very challenging postoperative feature to treat, even in cases without a significant diagnostic delay. The outcomes that are related to visual function are affected by the age of onset with better prognosis for infantile PCG than new-born PCG (26). Although the surgical treatment can provide acceptable control of IOP, only one-third of the patients will obtain good long-term visual acuity [15,26,27] Amblyopia is the leading cause of blindness followed by glaucomatous optic atrophy, visual deprivation nystagmus, and corneal opacity [26]. Treating PCG is always challenging and it is not finished with the surgery, long term follow-ups are mandatory.

The present study failed to establish a prognostic association between the outcome of IOP at the last follow-up and age at the time of diagnosis, gender, laterality, symptoms, corneal diameter, IOP before surgery, or diagnostic delay. Despite the fact that several studies have claimed the impact of corneal diameter and initial IOP to worsen the prognosis, others have not [1,28,29]. In this study, axial length could help in predicting the outcome of IOP control. However, a low number of eyes examined should be taken into consideration. When analysed within the scope of the Latvian population, PCG remains a challenging diagnosis, due to its rare distribution. Collaboration between healthcare providers could improve the diagnostic pathway.

## 5. Strengths and Limitations

The report provides a long-term follow-up period for surgically treated patients with PCG. In addition, all of the examinations were performed by the same ophthalmologist, and the patients underwent glaucoma surgeries performed by the same glaucoma specialist. Furthermore, this study demonstrates the incidence of PCG among the population of Latvia and provides evaluations of the outcomes.

However, several limitations should be addressed. Firstly, the study had a retrospective design. According to the statistics, the number of cases per year ranges from 0 to 3; therefore, the possibility of conducting a prospective study with a high number of participants is limited. Secondly, the small number of cases observed during the follow-up period could cause bias, regardless of the fact that the study utilised data that were collected over 15 years. Further studies, including those with an extended follow-up period, would be necessary to make more reliable conclusions in terms of the specific population.

## 6. Conclusions

During the 15-year follow-up period, the incidence of PCG in Latvia was 5.3 patients per 100,000 live births, which is similar to the situation in other European populations. PCG was more common among males than females, with a relative risk of 1.3. The enlargement of an eyeball was the leading finding that was reported by parents and guardians. Surgical treatment reflected an acceptable control for IOP in the majority of cases. 

## Figures and Tables

**Table 1:** Laterality, incidence, and relative risk related to gender.

Gender	Number	Bilateral Disease	Unilateral Disease	Number of Live Births	Incidence per 100,000	Relative Risk
Female	8	5	3	166,636	4.8	*
Male	10	1	9	175,962	5.7	1.3
Total	18	6	12	342,598	5.3	

* the reference group.

**Table 2:** The age at defining diagnosis and diagnostic delay according to laterality of the disease.

	<1 Month	≥1 Month to 3 Months	≥3 Months to 6 Months	≥6 Months to 12 Months	≥12 Months
**The age at defining diagnosis**	16.7% (3)	0%	16.7% (3)	61.1% (11)	5.6% (1)
Unilateral	25.0% (3)	0%	8.3% (1)	66.7% (8)	0%
Bilateral	0%	0%	33.3% (2)	50.0% (3)	16.7% (1)
**The diagnostic delay**	27.8% (5)	27.8% (5)	16.7% (3)	27.8% (5)	0%
Unilateral	25.0% (3)	25.0% (3)	16.7% (2)	33.3% (4)	0%
Bilateral	33.3% (2)	33.3% (2)	16.7% (1)	16.7% (1)	0%

**Table 3:** Signs and symptoms at the time of diagnosis and objective findings from examination under general anaesthesia.

The First Signs and Symptoms	Eyes % (Number)
Enlargement of an eyeball	75% (18)
Tearing	58.3% (14)
Opacity of cornea	41.7% (10)
Photophobia	29.2% (7)
Signs of lacrimal system inflammation	20.8% (5)
**Objective findings**	**Value**
Corneal diameter	Median 12.5 mm (IQR 1)
Axial length	22.4 ± 2.3 mm (range 19 to 28)
Intraocular pressure before surgery	27.4 ± 4.1 mmHg (range 22 to 35)

IQR—interquartile range.

**Table 4:** Intraocular pressure (IOP) values during follow-up.

Time	IOP mmHg	*p* Value *
Before surgery	27.4 ± 4.1	-
After surgery	16.9 ± 5.9	*p* < 0.001
After 3 months	16.3 ± 5.8	*p* = 0.63
After 6 months	16.2 ± 6.2	*p* = 0.86
After 12 months	15.0 ± 3.4	*p* = 0.26
At the last follow-up	14.6 ± 4.9	*p* = 0.67

* *p* value reflects difference between previous measurement.

**Table 5:** Incidence of primary congenital glaucoma (PCG) per 100,000 live born.

Authors	Population	Incidence per 100,000 Live Born Children
Pedersen KB et al., 2020 [7]	Denmark	4.80
Lee SJ et al., 2020 [13]	South Korea	11.0
Zetterberg M et al., 2015 [12]	Sweden	4.30
Alanazi FF et al., 2013 [14]	Saudi Arabia	40.0
Aponte EP et al., 2010 [2]	The United Sates (Minnesota)	1.46
Papadopoulos M et al., 2007 [1]	United Kingdom	5.41
Papadopoulos M et al., 2007 [1]	The Republic of Ireland	3.31
Taylor RH et al., 1999 [15]	Canada	8.0
Bermejo E et al., 1998 [16]	Spain	2.85
Gencik A, 1989 [17]	Slovakia (Gypsy population)	80.0
Gencik A, 1989 [17]	Slovakia (non-Gypsy population)	4.50

## Data Availability

The data presented in this study are available on request from the corresponding author, through the institutional review board. The data are not publicly available due to restrictions of the institution.
